# Solution-processed, semitransparent organic photovoltaics integrated with solution-doped graphene electrodes

**DOI:** 10.1038/s41598-020-77012-2

**Published:** 2020-11-17

**Authors:** Jan-Kai Chang, Yu-Yun Huang, Ding-Lun Lin, Jieh-I. Tau, Ting-Hao Chen, Mei-Hsin Chen

**Affiliations:** 1 Graduate Institute of Photonics and Optoelectronics and Department of Electrical Engineering, National Taiwan University, Taipei 106, Taiwan; 2 Department of Electro-Optical Engineering, National Taipei University of Technology, Taipei 106, Taiwan

**Keywords:** Materials science, Optics and photonics, Physics

## Abstract

In this work, by applying a transfer method simultaneously with a solution doping process for graphene as top electrodes, we demonstrate a solution-processed semitransparent organic photovoltaics (OPV). The work function of doped graphene under various doping conditions was investigated via photoemission spectroscopy. The transparent device was fabricated using PEDOT-doped graphene as electrodes, which provide an energetically favorable band alignment for carrier extractions. The solution-processed semitransparent organic photovoltaics exhibit the power conversion efficiency (PCE) of 4.2%, which is 85.7% of the PCE of control devices based on metallic reflecting electrodes, while maintaining good transparency at most visible wavelengths.

## Introduction

Since the beginning of the rapid development of organic semiconductors, extensive study of photoactive compounds and conductive polymers has led to promising applications in electronic devices such as sensors, memory, displays, lighting and solar cells with large processing areas and incorporating flexible technology^1–7^. In particular, numerous state-of-the-art achievements employing organic conductive thin films offer the distinguishing feature of semitransparency for organic optoelectronics, enabling novel applications such as wearable electronics, transparent display screens, synthetic skin, electrochromic windows and solar shingles to be integrated into everyday life^8–10^.

He intrinsic semitransparency and flexibility of films formed by organic molecules facilitates the development of organic optoelectronics that are transparent and can be integrated with curved surfaces. For instance, photoactive bulk heterojunctions (BHJs) consisting of poly(3-hexylthiophene)/phenyl-C61-butyric acid methyl ester (P3HT/PCBM) are intrinsically deep purple in color since the optical bandgap of P3HT is approximately 1.9 eV, which makes the heterojunction almost transparent to near-infrared radiation (> 650 nm)^11^. However, most conventional organic photovoltaics (OPVs) consist a nontransparent metal electrode, which can reharvest the reflected photons not absorbed in the first pass and thereby to improve the power conversion efficiency (PCE)^12^. The use of metal electrodes limits the transparency of the whole device and hence the feasibility of window-integrated applications is hindered.

To overcome obstacles to adapting innovative transparent elements to daily life, alternatives to transparent conductive electrodes have been intensively studied, including conductive polymers, various metal oxides, ultrathin metal layers or metal nanowires, carbon nanotubes, and photonic crystals^6,8,10,13–15^. Of additional interest are graphene transparent electrodes, which have been recognized as a next-generation substitute for indium tin oxide (ITO) due to their flexibility, high transparency, superior carrier mobility and excellent mechanical strength^16–20^. A great number of reports therefore suggest that graphene is an ideal candidate for achieving both adaptability and transparency for transparent facade integration^21–24^.

Nevertheless, neither tape detachment nor conventional polymer-assisted transfer is suitable for transferring a graphene top electrode onto organics, since these processes involve mechanical strains, solvent treatments, and residue issues that inevitably damage the underlying organic thin film^25,26^. In one study, stacked graphene layers were used as the anode for an inverted P3HT:PC_60_BM solar cell structure, where 10 layers of graphene were needed to achieve a PCE of 2.5%^27^. Thus, it has yet to be reported that graphene-based OPVs have simultaneously achieved semitransparency while yielding a PCE comparable to that of OPVs whose electrodes are deposited directly on the active layer.

In the present work, by using a polymer-free transfer method with an in situ doping process for graphene top electrodes, we demonstrate the fabrication of a promising graphene top electrode; the optimum performance of the device was examined by tuning the number of graphene layers as well as the active layer thickness. A systematic study of doped graphene under various doping conditions was completed via photoemission spectroscopy. The device we propose uses a PEDOT-doped graphene sandwich structure as a top electrode, which provides an energetically favorable band alignment to attain optimal efficiency while maintaining high transparency at most visible wavelengths. A solution-based process for fabricating semitransparent OPVs was therefore developed to facilitate vacuum-free technologies such as roll-to-roll processing. This result allows the development of semitransparent OPVs that exhibit approximately 50% visible light transmission and approximately 90% of the PCE of control devices based on metal reflecting electrodes, thereby providing a feasible green energy source and the additional advantages of saved time, less energy consumption and mass production for the solar industry.

## Results and discussion

To make a graphene top electrode capable of overcoming the aforementioned challenges, the transfer of unblemished high-quality graphene sheets is needed—this process was recently unveiled in our preceding work, offering excellent electrical properties using a polymer-free transfer method^28^. Moreover, graphene transferred by this method can be easily developed into stacked films in a layer-by-layer (LBL) structure to enhance the extrinsic conductivity, whereby the sheet resistance can be tuned to the requirements for functioning as an electrode. BHJ solar cells incorporating such graphene stacks as bottom transparent electrodes were first devised in the standard structure of glass/graphene/PEDOT:PSS/active layer/BCP/Al to investigate the adaptability of a polymer-free transferred graphene anode substituted for an ITO anode. Two common types of polymer donors, including P3HT and poly{[4,8-bis-(2-ethyl-hexyl-thiophene-5-yl)-benzo[1,2-b:4,5-b’]dithiophene-2,6-diyl]-alt-[2-(2′-ethyl-hexanoyl)-thieno[3,4-b]thiophen-4,6-diyl]} (PBDTTT-C-T), were selected to ensure that the obtained PCE was not unique to a specific type of high-performance semitransparent OPVs. The fabrication details are described in the experimental section, and all device performance parameters presented throughout the work were characterized in a nitrogen-filled glovebox.

**Figure 1:**
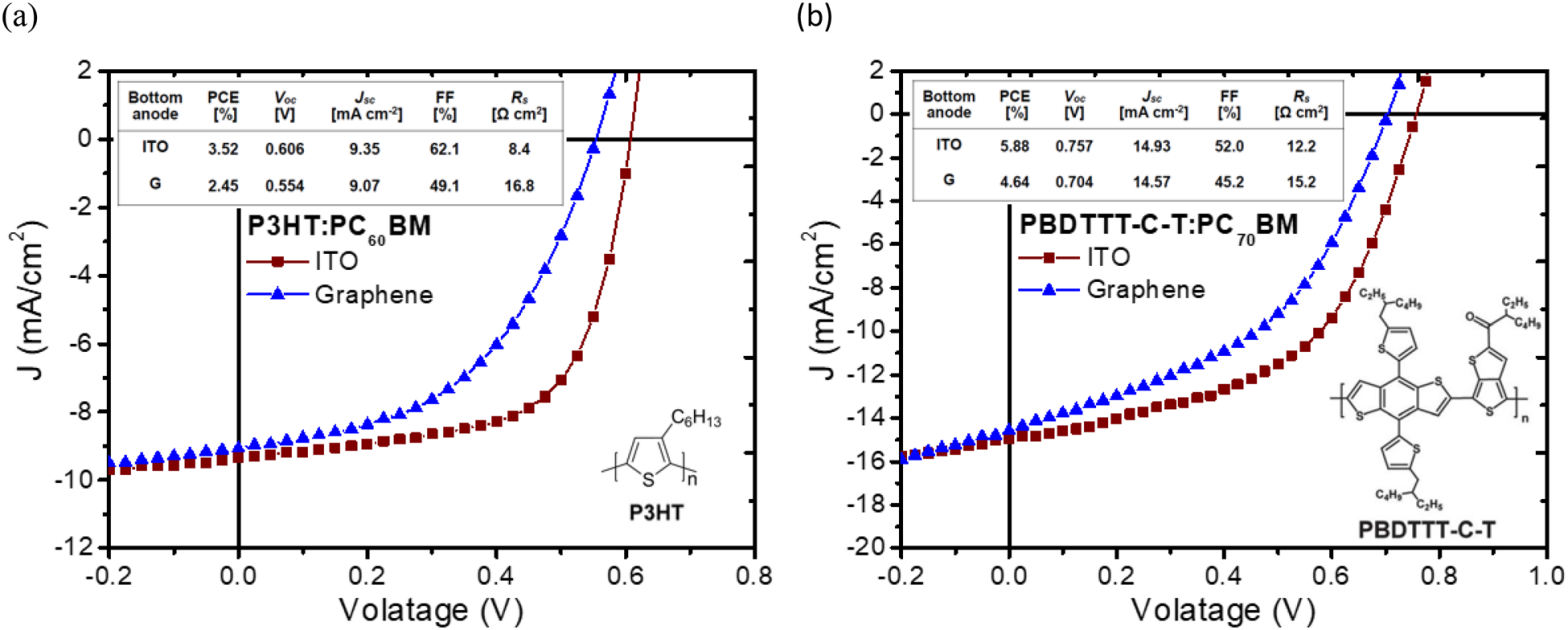
*J–V* characteristics of OPV devices using graphene and ITO bottom anodes for (**a**) P3HT:PC_60_BM and (**b**) PBDTTT-C-T:PC_70_BM BHJ as the active layer based on the structure of Anode/PEDOT:PSS/active layer/BCP/Al. The cell architecture of the standard structure and chemical structure of the photoactive polymer are shown as insets. Solar cell testing was performed inside a nitrogen-filled glovebox under simulated AM 1.5G irradiation (100 mW cm^−2^).

Figure 1 shows the current density–voltage (J–V) characteristics for a device using pristine graphene as the anode compared with a control device with an ITO anode based on the structure of Anode/PEDOT:PSS/active layer/BCP/Al. The observed rectification behavior in the J–V curve suggests that the graphene stacks transferred by the polymer-free method are capable of replacing ITO for electrode applications, although the graphene-based devices exhibit slight performance degradation in the open-circuit voltage (Voc), short circuit current (Jsc), fill factor (FF) and PCE for both P3HT and PBDTTT-C-T devices. Compared to a control device, the percentages of decreasing in Voc of the devices with graphene as the bottom anode is larger than that in Jsc as shown in Table 1. This degradation in photovoltaic parameters was attributed to the energy barrier at the anode/organic interface, since the work function of graphene (4.5 eV) is relatively smaller to facilitate carrier extraction, in contrast with that of UZO-treated ITO (4.9 eV)^29^. Nevertheless, the optimal PCEs of 2.45% (for the P3HT device) and 4.64% (for PBDTTT-C-T) for graphene-based OPVs indicate that the strategy proposed for fabricating graphene bottom anodes is feasible without polymer assistance or additional processes. Moreover, for both the devices using two different polymers as the active layer, the series resistance of the devices with graphene as the bottom anode are larger than that with an ITO anode. The series resistance arises from bulk resistance of the organic material, contact resistance at the metal–semiconductor, and resistance of the cathode. Since the sheet resistance of a graphene is normally larger than that of an ITO conducting thin film, which will result in a larger series resistance value in both polymer devices.

**Table 1:** Photovoltaic parameters of BHJ solar cells using ITO and graphene as transparent anodes in the standard structure (denoted as ITO and G, respectively).

Photoactive polymer	Bottom anode	PCE (%)	*V*_*oc*_ (V)	*J*_*sc*_ (mA cm^−2^)	FF (%)	*R*_*s*_ (Ω cm^2^)
P3HT	ITO	3.52	0.606	9.35	62.1	8.4
	G	2.45	0.554	9.07	49.1	16.8
PBDTTT-C-T	ITO	5.88	0.757	14.93	52.0	12.2
	G	4.64	0.704	14.57	45.2	15.2

**Figure 2:**
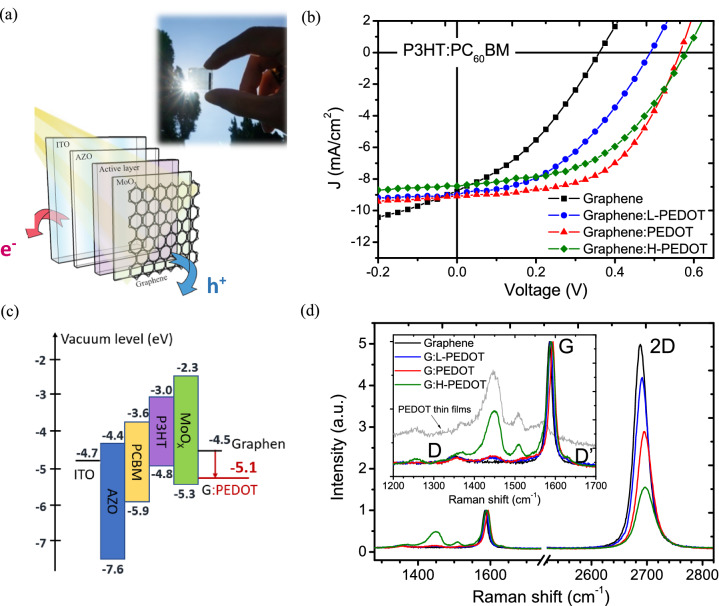
(**a**) Schematic illustration of a graphene-based semitransparent OPV and its real appearance. (**b**) *J–V* characteristics of P3HT:PC_60_BM BHJ solar cells using a PEDOT-doped graphene anode at various doping levels, such as-transferred graphene (G), PEDOT-doped graphene with light doping (G:L-PEDOT), optimal doping (G:PEDOT), and heavy doping (G:H-PEDOT). (**c**) Energy band alignment of the P3HT:PC_60_BM BHJ solar cell^32–34^. Note that the ionization energies measured from UPS are in eV. (**d**) Doping-dependent Raman spectral evolution of the PEDOT-doped graphene monolayer. The inset shows details of the G-, D-, and D’-bands, and the excess spectral feature between 1350 cm^−1^ and 1550 cm^−1^ that only appeared in the doped graphene is the Raman mode of the PEDOT:PSS dopant.

Using the transfer process we developed, transferring graphene stacks onto delicate organic thin films is feasible since polymer-free transfer is free from chemical and mechanical treatments such as solvent cleaning or the use of a stamping press. We further experimentally established an inverted OPV fabrication process to obtain the rarely achieved result of a graphene-based device with a graphene top electrode. Devices with a structure of ITO glass/AZO/P3HT:PCBM/MoOx were prepared as target substrates, after which polymer-free graphene transfer was performed to complete the inverted OPV (Fig. 2a).

As seen from Table 2 and Fig. 2b, the pristine graphene (G), PEDOT:PSS-doped graphene with light doping (G:L-PEDOT), optimal doping (G:PEDOT), and heavy doping (G:H-PEDOT) are prepared for comparison. The graphene-based semitransparent cell with pristine graphene stacks (black curve) yielded a Voc of 0.361 V and a PCE of 1.11%, which do not match the saturated Voc of ~ 0.6 V and PCE of ~ 3% for a typical inverted P3HT:PCBM BHJ solar cell^30,31^. Here, a PEDOT doping process was introduced in situ by adding PEDOT aqueous solution during transfer to modify the Fermi energy of the graphene stacks, achieving an energetically favorable band alignment (Fig. 2c) for hole extraction, where the energy of highest occupied molecular orbitals (HOMO) and lowest unoccupied molecular orbitals (LUMO) of P3HT and PCBM can be found in previous literatures^32–34^. In this case, the doped graphene stacks at various doping levels led to significant performance improvements in Voc, short-circuit current density (Jsc) and FF (Fig. 2b).

**Table 2:** Photovoltaic parameters of BHJ solar cells using PEDOT-doped graphene with different doping levels as the top electrode.

Top anode	Work function (Ev)	PCE (%)	*V*_*oc*_ (V)	*J*_*sc*_ (mA cm^−2^)	FF (%)
G	4.50	1.11	0.361	8.78	35.2
G:L-PEDOT	4.65	1.88	0.490	8.98	43.0
G:PEDOT	4.90	2.80	0.565	9.09	54.5
G:H-PEDOT	5.10	2.38	0.580	8.45	48.9

To gain insight into the doping effect, the doping-dependent evolution of the Raman spectral characteristics for the 2D-, G- and D-bands of doped graphene monolayers was studied, as shown in Fig. 2d. It was verified that the corresponding e–h coupling upon doping changes the Raman mode of graphene, such that the effect of increased carrier interaction decreased the intensity ratio I(2D)/I(G). The stiffening and sharpening of the G band with increasing doping level correspond to the nonadiabatic removal of Kohn anomalies at the BZ center (Γ) and the Pauli blocking of phonons decaying into e–h pairs, as reported previously^35^. Above all, the appearance of the D’ band in doped graphene indicates defect scattering and additional intravalley phonon emission from the dopants^36^. These phenomena imply that the presence of PEDOT molecules modified the carrier concentration of graphene, developing p-type conductivity to lower the Fermi level below the charge neutral point (i.e., the Dirac point).

**Figure 3:**
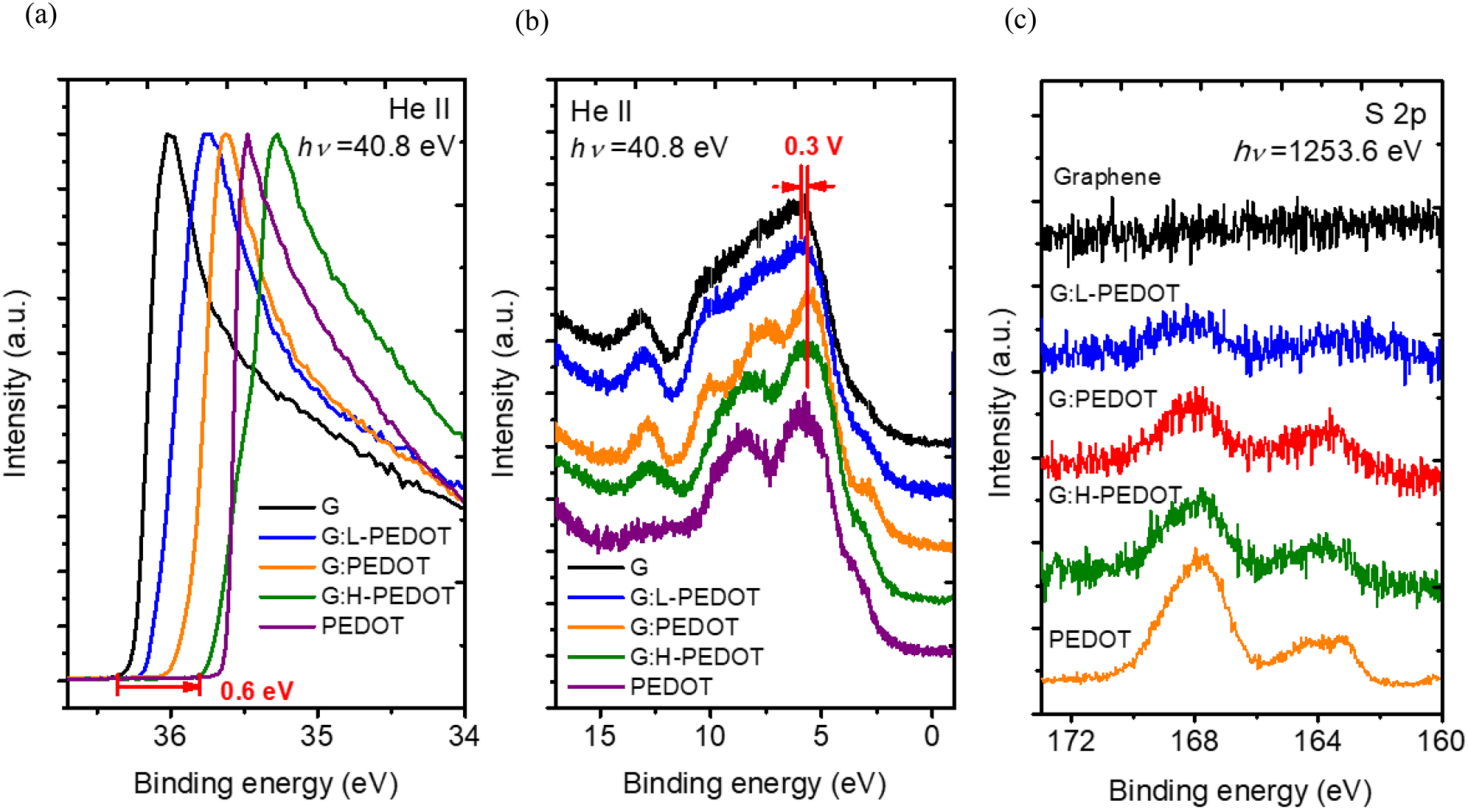
(**a**) UPS spectra near the onset region, (**b**) UPS spectra near the valence band, and (c)XPS spectra of the sulfur 2p core level of graphene with various doping levels, and the pristine PEDOT thin film.

To realize the energy alignment, ultraviolet photoemission spectroscopy (UPS) is used to measure the work function of the various doped graphene. The photon energy of 40.8 eV were utilized to excite the electrons in the materials and photoelectrons were collected with a monochrometer. All the binding energies in Fig. 3 are with respect to the Fermi level. The work function can be calculated from the onset of UPS spectra, which represent the vacuum level, and the Fermi level. Figure 3a shows that the onset of the spectra shift toward lower binding energy by 0.6 eV upon doping, indicating that the work function of graphene is increased by 0.6 eV. The spectra of valence-band shown in Fig. 3b shifts toward lower binding energy after the doping process. Since the zero point is the Fermi level, the shift of valence-band toward the Fermi level means the graphene films are p-doped. From X-ray photoemission spectroscopy (XPS), the effective doping ratios from 1.7% to 10.2% are measured by the intensity, corrected with sensitivity factor, of sulfur signal because sulfur atoms are only from PEDOT:PSS molecules, as shown in Fig. 3c. These results imply that the PEDOT:PSS molecules can enhance the doping effect by increasing the hole concentration in graphene and consequently taking the Fermi energy away from the Dirac point, resulting in a tunable work function from 4.5 eV to 5.1 eV (Table 2) for PEDOT-doped graphene. Such a gain in work function benefits device performance by diminishing the energy band mismatch, particularly at the MoOx/graphene anode interface, since this is precisely the spatial region where the photogenerated carriers are collected by the electrode.

**Figure 4:**
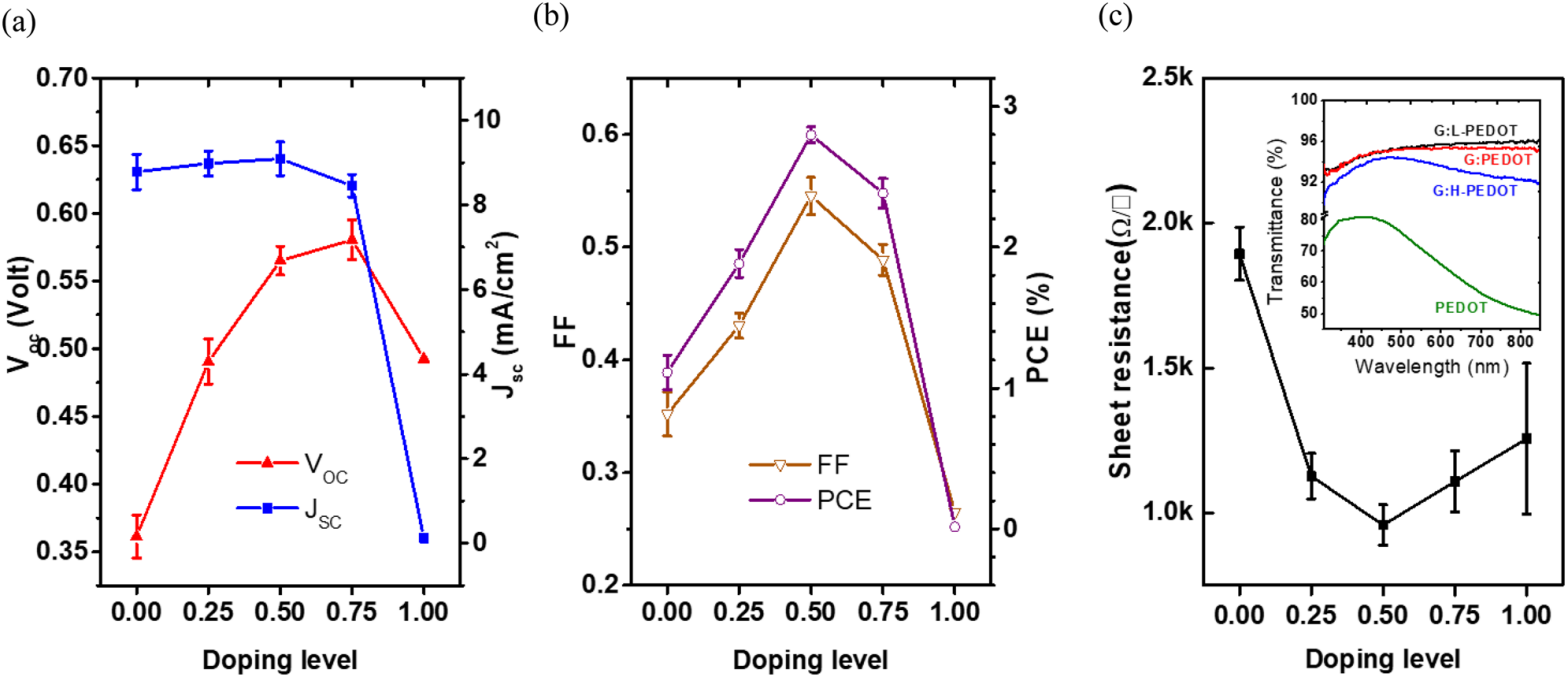
Evolution of photovoltaic parameters, (**a**) Voc, Jsc (**b**) FF and PCE, for P3HT:PC_60_BM BHJ solar cells incorporating a PEDOT-doped graphene anode with different doping levels. (**c**) Sheet resistance of PEDOT-doped graphene on glass substrates with different doping levels. The inset in (**c**) shows the transmittance for G:L-PEDOT, G:PEDOT, G:H-PEDOT and PEDOT for reference. Note that the doping levels of 0, 0.25, 0.5, 0.75 and 1 shown in the abscissa represent G, G:L-PEDOT, G:PEDOT, G:H-PEDOT and PEDOT, respectively.

The doping-dependent photovoltaic parameters for graphene-based devices are further demonstrated in Fig. 4, where an evident improvement in Voc up to 0.58 V can be achieved with optimal doping. This result again implies that the modified Fermi level of PEDOT-doped graphene indeed reduces the energy barrier at the organics/graphene anode to facilitate carrier transport and forms an energetically favorable band alignment for efficient hole extraction. However, the decline in Jsc, FF and PCE for the device using the heavily doped graphene (denoted as G:H-PEDOT) anode implies that excessive doping is detrimental to device performance owing to aggravated impurity and defect scattering, leading to larger sheet resistance for the overdoped G:H-PEDOT than the optimally doped G:PEDOT (Fig. 4c). In addition, the doping-dependent evolution of the valence-band structure (Fig. 3) reveals that the feature states of graphene were gradually transferred to those of PEDOT with an apparent depression of the 2p π state under heavy doping. Therefore, both overdoped impurities and π-electron efficiency decrease the sheet conductivity of graphene in the presence of an excess of PEDOT molecules, which degrades Jsc for devices with an overdoped graphene anode. It is also worth noting that the exterior color of the G:H-PEDOT sheets changed to dark gray, rendering the G:H-PEDOT devices opaque, as well as decreasing the transmittance, as can be observed for overdoped G:H-PEDOT in the inset of Fig. 4c.

**Figure 5:**
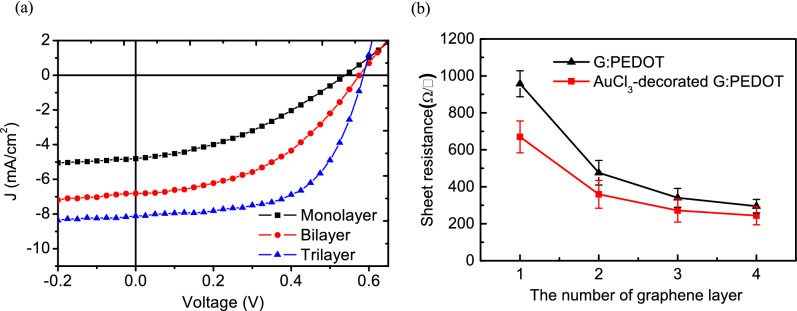
(**a**) J–V characteristics of semitransparent P3HT:PCBM inverted solar cells using monolayer, bilayer and trilayer AuCl3-decorated G:PEDOT as the top anode. (**b**) Sheet resistance of AuCl_3_-decorated G:PEDOT consisting of various LBL structures on glass substrates. Note that graphene sheets were doped with PEDOT during polymer-free transfer before being coated by AuCl_3_.

To further improve the performance of the semitransparent OPVs, the incorporation of AuCl_3_ was developed as a surface modification to achieve high-conductivity graphene sheets^37^. Since AuCl_3_ degrades graphene very quickly, it was spin-cast onto the prepared graphene stacks immediately before the J–V and external quantum efficiency (EQE) measurements to prevent degradation. The OPVs using AuCl_3_-decorated G:PEDOT stacks for the top transparent anode provided a better Voc with incremental improvements corresponding to the number of graphene stacks, as shown in Fig. 5a and Table 3, towards a saturation value of 0.6 V, with a PCE from 0.96% to 2.82%. This significant improvement came about because the natural defects and disorders in the CVD-grown graphene monolayers were gradually screened by the addition of further layers, thereby diminishing the shunt recombination of photogenerated carriers and consequently improving Jsc.

**Table 3:** Photovoltaic parameters of BHJ solar cells using different numbers of layers of AuCl3-decorated G:PEDOT sheets as the top electrode.

Electrode structure	PCE (%)	*V*_*oc*_ (V)	*J*_*sc*_ (mA cm^−2^)	FF (%)	*R*_*s*_ (Ω cm^2^)
Monolayer	0.96	0.539	4.80	37.1	61.0
Bilayer	1.76	0.576	6.81	44.8	33.3
Trilayer	2.82	0.585	8.11	59.4	13.4

Moreover, Fig. 5b shows that the sheet resistance reduction as a function of G:PEDOT layers is more experimental evidence for the improved performance of devices using G:PEDOT stacks as anodes. It is noteworthy that the presence of the dark AuCl_3_ film further hindered the transparency of the G:PEDOT sheets, and the transmittance of AuCl_3_-decorated quadrilayer G:PEDOT sheets was less than 60% at λ > 650 nm (not shown here). Hence, opaque quadrilayer (or more) G:PEDOT is not applicable to transparent electrode applications, although its conductivity improved with increasing numbers of layers.

**Figure 6:**
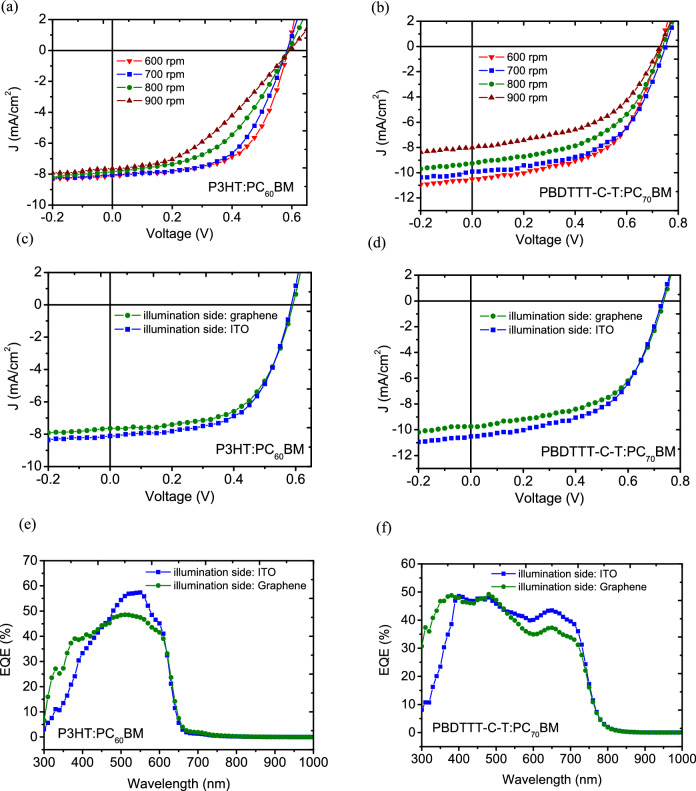
J–V characteristics for semitransparent OPVs using AuCl_3_-decorated G:PEDOT anodes with different thicknesses of (**a**) P3HT:PC_60_BM and (**b**) PBDTTT-C-T:PC_70_BM active layers. (**c**) J–V characteristics measured from both sides of semitransparent P3HT:PC_60_BM and (**d**) PBDTTT-C-T:PC_70_BM BHJ solar cells with a AuCl_3_-decorated G:PEDOT top anode. (**e**, **f**) are the external quantum efficiencies obtained in the same way as described in (**e**, **d**), respectively.

Figure 6a,b show the J–V characteristics for semitransparent OPVs with various spinning rates in coating P3HT:PC_60_BM (Table 4) and PBDTTT-C-T:PC_70_BM (Table 5) active layers for performance optimization. Not surprisingly, an enhancement in Jsc for both photoactive blends was achieved by increasing the thickness of the active layers, as more charge carriers can be generated by extending the optical path to result in more photons absorbed by a thicker film. This enhancement occurred in addition to an evident improvement in FF, since an active layer with sufficient thickness prohibits water permeation during the wet-transfer process, preventing water damage from diminishing the shunt paths developing within the water-sensitive organic films. Consequently, improved values of Rsh derived from the J–V characteristics can also be observed, as listed in Tables 4 and 5, leading to optimal PCEs of 2.82% and 4.20% for P3HT:PC_60_BM and PBDTTT-C-T:PC_70_BM semitransparent OPVs, respectively.

**Table 4:** Photovoltaic parameters of semitransparent P3HT:PC_60_BM devices with different thicknesses of the active layer.

Speed rate for active layer deposition	PCE (%)	*V*_*oc*_ (V)	*J*_*sc*_ (mA cm^−2^)	FF (%)	*R*_*sh*_ (Ω cm^2^)
900 rpm	1.80	0.593	7.64	39.7	513.2
800 rpm	2.21	0.587	7.86	48.0	744.8
700 rpm	2.66	0.583	8.07	56.7	1041.3
600 rpm	2.82	0.585	8.11	59.4	1409.0

**Table 5:** Photovoltaic parameters of semitransparent PBDTTT-C-T:PC_70_BM devices with different thicknesses of the active layer.

Speed rate for active layer deposition	PCE (%)	*V*_*oc*_ (V)	*J*_*sc*_ (mA cm^−2^)	FF (%)	*R*_*sh*_ (Ω cm^2^)
900 rpm	2.90	0.729	8.01	49.7	429.2
800 rpm	3.48	0.739	9.26	51.0	432.5
700 rpm	4.01	0.751	9.91	53.6	457.7
600 rpm	4.20	0.731	10.51	54.4	507.9

For a better understanding of doped graphene in electrode applications, the semitransparent OPVs were illuminated on each side under an AM1.5G solar light to demonstrate semitransparency with green power generation. From Fig. 6c–f, the J–V and EQE characteristics demonstrate that incident light was able to pierce the whole device, a distinguishing defining feature of transparency. Table 6 summarizes the photovoltaic parameters measured from the respective sides of the semitransparent OPVs shown in Fig. 6c–f. Regardless of the exposed side, the P3HT:PC_60_BM semitransparent OPVs exhibited decent performance in all photovoltaic parameters—Voc (~ 0.59 V), FF (59%), and PCE (over 2.6%)—while the values for the PBDTTT-C-T:PC_70_BM devices were ~ 0.73 V, 54% and over 3.9%, respectively. Only a small variation in Jsc is associated with the side where the light entered, leading to a < 6% difference in PCE when the devices are illuminated from the AuCl_3_-decorated G:PEDOT and ITO electrodes. The slightly worse of PCE of semi-transparent solar cells, as compared to regular non-transparent solar cells, are because of lack of back reflection from reflective electrodes and higher resistance of doped graphene. Nonetheless, this semi-transparent solar cell, with average transmittance at visible light of 40%, gains the flexibility when using it to window-integrated application.

**Table 6:** Photovoltaic parameters of semitransparent OPVs illuminated on the ITO and graphene sides.

Photoactive polymer	Illumination side	PCE (%)	*V*_*oc*_ (V)	*J*_*sc*_ (mA cm^−2^)	FF (%)
P3HT	ITO	2.82	0.585	8.11	59.4
	Graphene	2.67	0.590	7.64	59.2
PBDTTT-C-T	ITO	4.20	0.731	10.51	54.4
	Graphene	3.95	0.736	9.74	54.7

Figure 6e,f reiterate the spectral difference between the EQE spectra of AuCl_3_-decorated G:PEDOT and ITO; the device illuminated from the graphene side reached a higher EQE at short wavelengths (> 450 nm) than that of the device illuminated from the ITO side, but the relative trend was reversed at long wavelengths regardless of which photoactive blends were used. This behavior not only agrees well with the decreased transmittance at long wavelengths for the G:PEDOT transmission spectrum (the inset of Fig. 4c) but is also consistent with reports of significant attenuation at short wavelengths when light traveled through an ITO/ZnO electrode^23^. Therefore, the slight PCE variation between devices illuminated on opposite faces can be attributed to the different light management processes of front electrodes, again suggesting that the optical and electrical properties of AuCl_3_-decorated G:PEDOT are comparable to those of conventional electrodes and can also achieve decent Voc, Jsc, FF and PCE values for photovoltaic devices.

**Figure 7:**
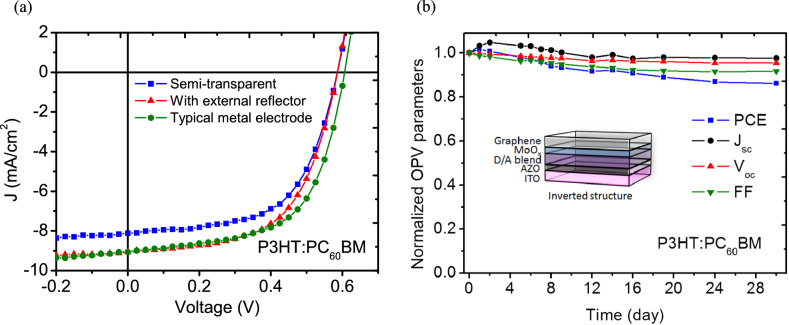
(**a**) J–V characteristics of typical and semitransparent P3HT:PC_60_BM OPVs with and without a back reflector. (**b**) Time evolution of photovoltaic parameters for a semitransparent device during the first 720 h after being fabricated.

As mentioned above, a major limitation to PCE in semitransparent OPVs is poor photon harvesting owing to the inevitable trade-off between capturing photons and transparency. Applying an external mirror is hence a flexible way to develop a solar window along with strong green power generation, as the photocurrent of a semitransparent device is expected to improve with more photogenerated carriers from the reflected photons and extended optical path. Accordingly, an evident increase in Jsc can be observed in Fig. 7a, which was achieved by establishing the cavity effect with the integration of a reflector, leading to a PCE of 3.11% for P3HT:PC_60_BM devices (Table 7). This high-performance graphene-based OPV attained approximately 95% of the PCE of a typical P3HT:PC_60_BM device using a metal electrode. Moreover, as illustrated in Fig. 7b, the proposed semitransparent P3HT:PC_60_BM device maintained at least 90% of the original values of its photovoltaic parameters after 720 h, indicating excellent stability when using the AuCl_3_-decorated G:PEDOT as the top electrode.

**Table 7:** Photovoltaic parameters of semitransparent OPVs with and without a back reflector.

Photoactive polymer	Reflector	PCE (%)	*V*_*oc*_ (V)	*J*_*sc*_ (mA cm^−2^)	FF (%)
P3HT	w/o	2.82	0.585	8.11	59.4
	w/	3.11	0.585	9.04	58.8
	Typical electrode	3.29	0.605	9.06	60.2

**Figure 8:**
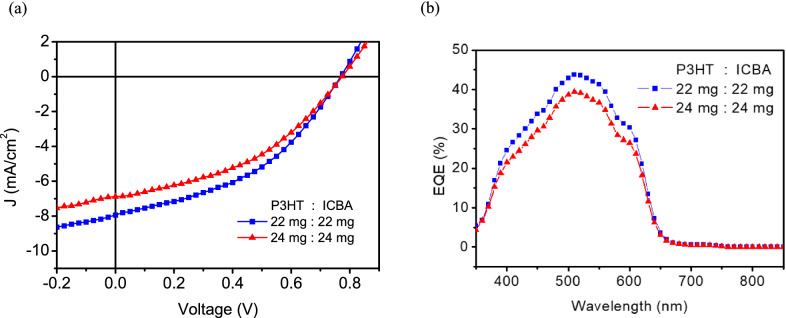
(**a**) J–V characteristics of semitransparent P3HT:ICBA OPVs using solution processing under various blend ratios of the P3HT:ICBA active layer (in 1 mL o-DCB). (**b**) EQE spectra of the P3HT:ICBA devices.

With the development of AuCl_3_-decorated G:PEDOT as the top electrode for OPVs, the goal of a solution-processed OPV is now one step away. Incorporating a solution-processed substitute for the hole transport layer (HTL), such as PEDOT:PSS or a transition metal oxide^38–40^, could replace thermally deposited MoOx to achieve vacuum-free fabrication. Here, the hydrophobic photoactive film was illuminated with UV light for 30 s before spin-coating with IPA-diluted PEDOT:PSS HTL to match surface energies and achieve better contact at the junctions. Additionally, a UV-resistant P3HT:ICBA photoactive blend was used to minimize polymer degradation during UV treatment. Here, various blend ratios of P3HT:ICBA in o-dichlorobenzene (o-DCB) were tested in solution-processed OPVs, as shown in Table 8 and Fig. 8. The optimal device with a blend ratio of 22 mg P3HT and 22 mg ICBA yielded a Voc of 0.77 V, Jsc of 7.94 mA/cm^2^ and FF of 42.4%, leading to a decent PCE of 2.60% for a solution processed device.

**Table 8:** Photovoltaic parameters of semitransparent OPVs using solution processing.

Photoactive blend	Blend ratio (mg)	PCE (%)	*V*_*oc*_ (V)	*J*_*sc*_ (mA cm^−2^)	FF (%)
P3HT:ICBA	22:22	2.60	0.769	7.94	42.4
	24:24	2.22	0.774	6.88	41.7

## Conclusion

We have demonstrated the fabrication and function of a semitransparent OPV with the PCE of PBDTTT-C-T:PC_70_BMPCE based solar cells of 4.2%, using p-doped graphene. The proposed doping process allows graphene to be integrated with arbitrary substrates, including delicate organic thin films. The energy-band alignment and electronic structure of the doped graphene were investigated in detail via Raman and photoemission spectroscopy to assess the doping effect, showing a tunable work function from 4.5 eV to 5.1 eV. Optimally doped graphene is therefore a very good alternative for a transparent conducting electrode and was successfully integrated with OPVs as the top anode, achieving semitransparency. By engineering the interface between graphene layers and beneath the HTL, we probed the doping-dependent characteristics of graphene-based OPVs systematically to verify that an energetically favorable band alignment is essential to performance improvement. As a result, the optimized PBDTTT-C-T:PC_70_BM OPVs not only featured semitransparency but also exhibited high stability with remarkable performance in Voc (0.73 V), FF (54%), and PCE (4.2%) compared to that of conventional electrodes using metal electrodes. Above all, a solution-processed OPV with decent power conversion efficiency has been demonstrated, indicating its potential as a time-saving, low-cost and lightweight technology. These results suggest that graphene electrodes are practical for transparent facade integration and thus feasible for application to electrical power generation sources in highly populated urban areas.

## Methods

### Polymer-free transfer with in situ doping of graphene

Commercial monolayer graphene films synthesized by CVD on copper foils were purchased from Graphene Supermarket^41^. The graphene/copper sheets of the desired size were placed into graphite confinement floating on an ammonium persulfate etchant (Alfa Aesar, 0.2 M) to remove the copper carrier. After the undesired copper foil was thoroughly etched, a blended aqueous solution (mixture of DI water and IPA at a ratio of 10:1) was subsequently substituted for the etchant^42^. At the same time, PEDOT:PSS solution was gradually introduced into the floating graphene film by liquid-phase diffusion. Then, the doped solution was rinsed off with the IPA-DI water mixture just before setting an arbitrary target beneath the doped graphene, ensuring that the immersed target substrate was free of PEDOT solution. Finally, the IPA-DI water mixture was pumped out to lower the doped graphene onto the target substrate, and the sample was heated at 70 °C for 10 min to improve adhesion. Note that graphene stacking in an LBL structure can be achieved by repeating this transfer process. The quality of the as-transferred graphene films was characterized by Raman spectroscopy, showing flawless planarity with almost no Raman D-band^28^.

### Fabrication of BHJ solar cells

The BHJ solar cells in this study were either ITO/AZO/P3HT:PC_60_BM/MoOx/graphene or ITO/AZO/PBDTTT-C-T:PC_70_BM/MoOx/graphene in an inverted structure. First, sol–gel AZO layers deposited on precleaned ITO substrates were prepared from a Zn precursor consisting of 0.5 M zinc acetate and monoethanolamine (MEA) in IPA with 1.35% aluminum nitrate in a molar ratio and subsequently spin on top of ITO substrate baked at 280 °C for 10 min to form the electron transport layer for an inverted BHJ solar cell^6,32^. Next, the following photoactive blends (Alderich, 1:1 with 2.5 wt% in o-DCB for P3HT:PC_60_BM; Alderich, 1:1.5 with 1.2 wt% in o-DCB and 4% additive of 1,6-diiodohexane for PBDTTT-C-T:PC_70_BM) were spin-cast in a nitrogen-filled glovebox from 600 to 900 rpm for 40 s. Then, a 15-Å MoOx interlayer was thermally deposited at a pressure below 10^−6^ Torr to form the HTL for the inverted solar cell. Moreover, a solution of PEDOT:PSS mixed with IPA (1:1 v/v) was spin-cast onto the sample at 4000 rpm for 40 s to complete the HTL for the vacuum-free process. Finally, the doped graphene sheet was transferred onto the as-fabricated ITO/AZO/active blend/HTL substrate using the polymer-free method with in situ PEDOT doping as mentioned above. Note that only the top surface of the graphene electrode was doped with AuCl_3_ (Alderich, 20 mM) before completion of the 10-mm^2^ graphene-based semitransparent OPVs.

### Material and device characterization

In this study, the J–V characteristics of the OPVs were measured in a glovebox under AM1.5G illumination with an irradiation intensity of 100 mW cm^−2^. The photoemission experiments were performed in a Phi5400 ultrahigh vacuum system with a base pressure of 10^−10^ Torr for XPS and UPS analyses. The photoelectrons excited by He II (hv = 40.8 eV) or Al Kα sources were collected using a hemispherical analyzer with an overall resolution of 0.05 eV.
